# Phages Shape Microbial Dynamics and Metabolism of a Model Community Mimicking Cider, a Fermented Beverage

**DOI:** 10.3390/v14102283

**Published:** 2022-10-17

**Authors:** Pierre Ledormand, Nathalie Desmasures, Margot Schlusselhuber, André Sesboüé, Jérôme Ledauphin, Marion Dalmasso

**Affiliations:** 1 Normandie University, UNICAEN, UNIROUEN, ABTE, 14000 Caen, France; 2 Normandie University, UNICAEN, LMNO, UMR CNRS 6139, 14000 Caen, France

**Keywords:** model microbial communities, phages, disturbance, fermented beverage, cider, phage–bacteria interactions

## Abstract

Model microbial communities are often studied to better understand interactions and fluxes during fermentation processes. However, models that take into account the potential impact of bacteriophages (phages), which are recognized as drivers of microbial communities, are scarce, especially in fermented foods. This study aimed at investigating the behavior of a cider model microbial community, which was subjected to disturbance in the presence or absence of phages and at two different temperatures (25 °C and 15 °C). The model microbial community was composed of three lactic acid bacteria (LAB) strains belonging to the species *Liquorilactobacillus mali*, *Leuconostoc mesenteroides* and *Oenococcus oeni*, and of a *Saccharomyces uvarum* yeast strain. Two phages were selected, targeting *L. mali* and *Ln. mesenteroides* strains. In order to follow the behavior of the microbial community model, the phages and microbial strains were enumerated at several time points, and the metabolic signatures (sugar consumption, production of organic acids and volatile organic compounds) of the model microbial community were monitored. At 25 °C, the community with phages (P) was significantly closer to the control condition (C) than to the condition without phages (D). Microbial levels were similar between conditions C and P, which were characterized by high concentrations of compounds such as 2-phenylethanol, ethyl octanoate and isoamyl alcohol, and more globally by a more complex metabolic signature than that of condition D. In condition D, *L. mali* and *Ln. mesenteroides* were dominant while *S. uvarum* and *O. oeni* were less present, and this condition was characterized by a high concentration of ethyl lactate. At 15 °C, condition P differed from conditions C and D, as *Ln. mesenteroides* was not detected while the other strains all reached approximately the same levels. The metabolic range of condition P was less important than for conditions C and D. The current study showed that the influence of phages on the model microbial community dynamics and metabolisms after a disturbance phenomenon was temperature-dependent.

## 1. Introduction

The fermentation of food products has been used for centuries to preserve and stabilize foods. Thus, many fermented foods and beverages have been developed and continue to be created worldwide. The diversity of fermented foods and beverages around the world is well reviewed [1,2]. Many works tend to demonstrate that microbial diversity is correlated to ecosystem functioning across different habitats and spatial scales [3]. The diversity and dynamics of microbial communities in a large variety of fermented foods are well described [4]. Within these communities, micro-organisms can be extremely responsive to environmental changes such as temperature variations, oxidative stress or chemical exposure such as xenobiotics, which can disturb microbial equilibria and result in an unbalanced state. This phenomenon is extensively described in the gut microbiota, for example, particularly, to try to explain the link with some diseases and dysbiosis [5,6]. In this context, the role of phages, the viruses of bacteria, still needs to be thoroughly investigated to evaluate their potential as drivers of microbial communities [7,8]. For example, they are known to contribute to reshaping the gut microbiota diversity after autochthonous fecal virome transplantation following antibiotic-based dysbiosis in a mouse model [9]. In fermented food and beverages, the studies of the impact of phages on the microbial communities are scarce and almost exclusively focused on the ability of phages, used alone or in cocktails, to destroy undesirable and pathogenic bacteria [10,11,12,13,14].

Model microbial communities are often constructed to better understand the functioning of a complex microbial community, especially in foods. These model microbial communities are often used to understand interactions and fluxes during fermentation processes, as is the case when studying the co-cultures of cheese starters [15] or bioprotective microbial communities [16,17]. Model microbial communities are also of interest to improve industrial processes, for example, when used to extend the shelf-life of a cooked meat product [18]. Model microbial communities have also been developed to improve food quality, for example, in the case of soy sauce, whose fermentation properties were improved by the co-cultures of *Bacillus subtilis* strains [19]. Microbial models in foods were also established and used to improve the production of specific metabolites such as vitamin C [20,21]. Concerning fermented beverages, few studies using model microbial communities are available. For example, yeast co-cultures have been tested to improve the aromatic profile of white wine Sauvignon blanc [22]. In the same way, the model microbial communities of lactic acid bacteria (LAB) and yeasts were developed to improve the probiotic properties of a fermented cereal beverage, boza [23]. To date, no model microbial communities are involved in studies of the role of phages in the modulation of microbial equilibria in fermented foods. This is especially true when it comes to fermented beverages, and more particularly to cider. Cider is an alcoholic fermented apple beverage with harsh physico-chemical conditions such as low pH values (3.0–4.0), and the presence of ethanol, polyphenols and volatiles [24,25]. The micro-organisms involved in cider fermentation are mainly yeasts (*Saccharomyces* and non-*Saccharomyces* species), performing the alcoholic fermentation, and LAB, i.e., *Oenococcus* sp., *Leuconostoc* sp., *Pediococcus* sp. and *Lactobacillus* sensu lato, performing the malolactic fermentation. Even if phage occurrence in cider seems to be low, their role still needs to be taken into consideration in this complex matrix [26,27]. The aim of the current study was to investigate the role played by phages on the behavior of a model microbial community inspired by cider. The creation of a disturbance in the model microbial community was used to assess the impact of phages on the microbial fluctuations. Thus, microbial dynamics and some metabolic fluxes were explored.

## 2. Materials and Methods

### 2.1. Bacteriophages, Bacterial Strains and Growth Conditions

Four microbial strains, *Liquorilactobacillus mali* UCMA 16447, *Leuconostoc mesenteroides* CIM 3004, *Oenococcus oeni* UCMA 19297 and *Saccharomyces uvarum* UCMA 10446, were used in this study to create a model microbial community. Phage UCMA 21115 targeting *L. mali* UCMA 16447 was used because it was the first lytic siphophage isolated from cider [27]. The lytic phage PLE001 targeting *Ln. mesenteroides* CIM 3004 was also used to complete the model, as *Ln. mesenteroides* is a species found in cider. Strains UCMA 16447, UCMA 19297, UCMA 10446 and the phage UCMA 21115 all originated from cider and came from the UCMA collection (Université de Caen Microbiologie Alimentaire, Caen, France). The strain CIM 3004 and its phage PLE001 originated from the dairy environment, and came from the FranceMIL national collection (CNIEL, Paris, France).

Bacterial strains were routinely grown in de Man Rogosa & Sharpe (MRS; Difco) broth adjusted to pH 5.5, which was supplemented with 5 g/L of fructose and 0.5 g/L of cysteine and incubated at 30 °C for 24 h or more. Phage propagations were carried out in MRS broth, which was supplemented with 10 mM of M_g_Cl_2_ and 10 mM of M_g_SO_4_. Briefly, host strains were grown until an OD_600nm_ value of 0.2, and then 100 µL of phage lysate (10^9^–10^10^ PFU/mL) was added, followed by an incubation at 30 °C until lysis occurred. The phage lysate was centrifuged at 4700× *g* for 20 min and filtered using 0.45 µm filters, then stored at 4 °C. Phage titration was performed using the double-layer plate technique in MRS (0.5% (*w*/*v*) agar), which was supplemented with 10 mM of CaCl_2_ and 10 mM of MgSO_4_ and incubated at 30 °C for 24 h to allow the formation of plaques. Phage titration was expressed in plaque-forming units per mL (PFU/mL).

### 2.2. Experimental Design

**Figure 1:**
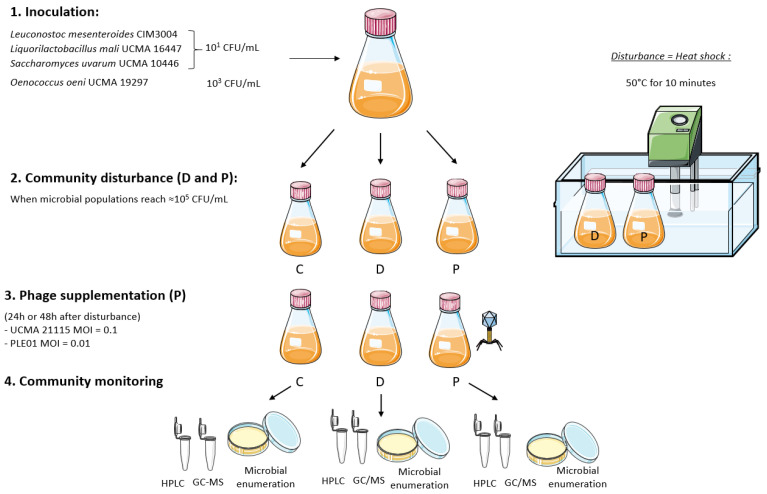
Experimental design of the study. The model microbial community was incubated at 25 °C or at 15 °C, and the control community (C), the community subjected to disturbance (D) and the community subjected to disturbance followed by phage addition (P) were monitored over time.

The global experimental design is summarized in Figure 1.

The four bacterial and yeast strains were separately grown overnight in MRS broth at 30 °C as pre-cultures. Then, 600 mL of fresh MRS broth adjusted to pH 5.5 was simultaneously inoculated at 10^1^ CFU/mL with each of the three strains *L. mali* UCMA 16447, *Ln. mesenteroides* CIM 3004, and *S. uvarum* UCMA 10446, and at 10^3^ CFU/mL with *O. oeni* UCMA 19297 strain, and incubated at 25 °C. After 24 h of incubation (when strains reached ~10^5^ CFU/mL), the 600 mL of co-culture was divided into three aliquots of 200 mL each. The first aliquot was used as control (C), while the two others (D and P) were subjected to a heat shock of 50 °C for 10 min, in order to create a disturbance of the microbial equilibria. The objective was to disturb the microbial equilibria without eliminating a microbial strain of the model. After the heat shock, the aliquots were immediately and rapidly cooled down to 25 °C, and incubation continued. Twenty-four hours after disturbance, phage UCMA 21115 and phage PLE001 were introduced at a MOI of 0.1 and of 0.01, respectively, into aliquot P only, in order to mimic the low abundance of phages in cider [26]. Preliminary tests of different MOIs were performed to determine the most suitable MOI to be used in this experiment (data not shown). Samples were taken from C, D and P every 24 h from inoculation, for at least 5 days, for pH measurements (Mettler Toledo, Colombus, OH, USA) and microbial and phage enumerations, and the HPLC and GC-MS analysis, as described below.

The same experimental design was repeated at 15 °C. Disturbance was performed in the same conditions when each strain had reached ~10^5^ CFU/mL (at 72 h). The phages were introduced into aliquot P at 120 h, i.e., 48 h after disturbance.

All the experiments were performed in biological triplicates.

### 2.3. Microbial Enumeration

For each sample, bacterial counting was performed on MRS agar medium (Difco, Franklin Lakes, NJ, USA), which was supplemented with 32 mg/L of 5-bromo-4-chloro-3-indolyl β-d-galactopyranoside (X-Gal; Fisher, Illkirch, France), 100 mg/L of pimaricin (Laboratoire Standa, Caen, France) and 7 mg/L of cycloheximide (Merck, Darmstadt, Germany) after 72–96 h incubation at 30 °C. On this medium, *Ln. mesenteroides* CIM 3004 colonies were the only ones that adopted a characteristic blue coloration. *L. mali* UCMA 16447 and *O. oeni* UCMA 19297 were distinguished by their morphological aspect: the *L. mali* colonies were rather big, bulging and creamy, while the *O. oeni* colonies were small. *Saccharomyces uvarum* UCMA 10446 was enumerated on Yeast Glucose Chloramphenicol (YGC; Biokar Diagnostics, Allonne, France) after 48 h of incubation at 25 °C.

### 2.4. Organic Acids and Ethanol Quantification by High Performance Liquid Chromatography (HPLC)

Reverse phase HPLC (Waters Alliance HPLC system with 2695 pump) was performed at 0.6 mL/min in an isocratic mode for 30 min using 5 mM of H_2_SO_4_ buffer as mobile phase. Separation was carried out with a Carbomix HNP5 8% cross-linking 7.8 × 300 mm column at 40 °C (Sepax Technologies, Guangzhou, China) and compounds were monitored by refractometry at 30 °C (2414 Refractive Index Detector; Waters, Milford, MA, USA). After centrifugation at 4700× *g* for 10 min, the supernatants of the different samples (C, D and P) were two-fold diluted with mobile phase before an injection of 20 µL. Ethanol and organic acid (acetic, lactic or citric) production as well as sugar consumption (fructose or glucose) were monitored by a comparison to the standards of each compound. The standards were injected at concentrations ranging from 2 mM to 64 mM.

### 2.5. Volatile Compounds Identification and Quantification Level by Gas-Chromatography Mass Spectrometry Analyses (GC-MS)

Volatile compounds were extracted from 1 mL of sample aliquot of supernatant according to a method developed in a previous study [28]. Briefly, all samples were spiked with 2 internal standards, 4-methylpentan-2-ol and ethyl undecanoate, both at a final concentration of 2 mg/L. Micro-extraction by Packed Sorbent (MEPS) was operated using a Barrel Insert and Needle (BIN) assembly filled with a C18 sorbent using 8 × 150 μL of spiked samples. Volatile compounds were recovered by elution with 50 μL of ethyl acetate. One microliter of the extract was injected with a 1/5 split ratio in a GC-MS Varian 3800 Gas Chromatograph coupled with a Varian Saturn 2000R Mass Spectrometer. Helium was used as the carrier gas with a 1 mL/min flow rate. The compounds were separated on a DB-5MS capillary column (60 m, 0.25 mm i.d., 0.25 μm film thickness, J&W Scientific, Santa Clara, CA, USA) with the following program temperature of the GC oven: 40 °C during 10 min, followed by a 5 °C-increase per min, and a final temperature of 240 °C held for 10 min. Volatile compounds were identified according to a procedure developed in previous works [29]. The peak areas of each volatile organic compound (VOC) were obtained using the most abundant and specific ion (*m*/*z* ratio) produced in electron ionization (EI) mode with the Mass Spectrometer. The relative areas were then calculated by dividing the peak areas by the peak area of ethyl undecanoate (internal standard). The quality control of the analyses was operated by comparing the areas obtained for the peaks of the two internal standards (ethyl undecanoate and 4-methylpentan-2-ol). The levels of each VOC were compared using the relative areas obtained between the three conditions.

### 2.6. Statistical Analysis

Statistical analyses of the microbial enumeration data were conducted using R software (v 4.1.2) and Prism (GraphPad, San Diego, CA, USA). The differences between C, D and P conditions were analyzed with a Kruskal–Wallis test followed by Dunn’s procedure for multiple comparisons, with a *p*-value adjusted with the Benjamini–Hochberg method. Concerning sugars, organic acids and VOC concentrations, statistical analyses were performed at each sampling time with the same method. A *p*-value of ≤ 0.05 was considered to be significant. A Principal Component Analysis (PCA) was performed using R software (v 4.1.2) and for each time of analysis, variables with a contribution superior to 3 and with a cos^2^ superior to 0.5 (for Dim 1 and Dim 2) were retained.

## 3. Results

### 3.1. Phages Have a Different Impact on the Microbial Dynamics Depending on the Temperature

**Figure 2:**
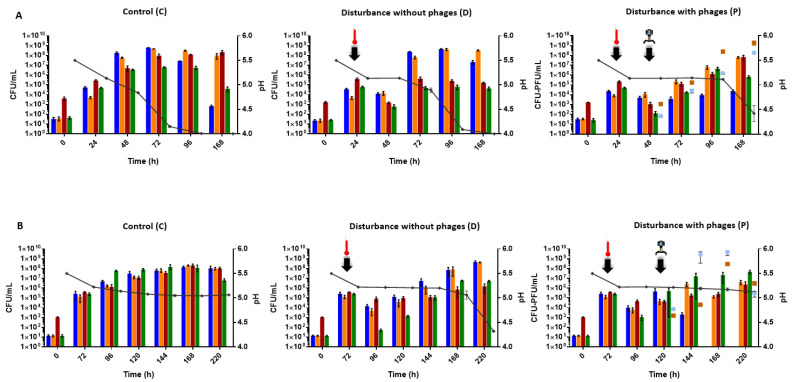
Dynamics of the microbial populations and pH (black curve) of the model microbial community in the three experimental conditions (control, disturbance without phages and disturbance with phages) at 25 °C (**A**) and at 15 °C (**B**). *Ln. mesenteroides* CIM 3004 is represented in blue, *L. mali* UCMA 16447 in orange, *O. oeni* UCMA 19297 in red and *S. uvarum* UCMA 10446 in green. Phage UCMA 21115 was added at a MOI of 0.1 and phage PLE001 at a MOI of 0.01, and are represented with orange squares and blue circles, respectively. The black arrows indicate heat shock and phage addition. The values were derived from biological triplicates and error bars represent standard deviations.

The model microbial community was designed to assess the effect of phages on the microbial equilibria after a disturbance phenomenon. First, the presence of prophages inside the bacterial genomes was checked. Only the genome of *L. mali* UCMA 16447 was available, and one complete prophage was found with PHASTER [30] (data not shown). The induction of potential prophages was checked using mitomycin C at 0.1 and 1 µg/mL, and no induction was observed for the three bacterial strains, suggesting that no inducible prophage was present (Figure S1). Thus, the microbial composition of the control community (C, without disturbance, without phage); of the community subjected to disturbance without phage addition (D); and of the community subjected to disturbance followed by phage addition (P), at 25 °C (Figure 2A) and at 15 °C (Figure 2B), was monitored over time.

#### 3.1.1. Disturbance Occurred in the Model Microbial Community after a Heat Shock

The first step of this work was to ensure that the heat shock applied to the model microbial community disrupted the initial microbial equilibria, and thus, generated a disturbance without completely eradicating any strain from the model. This was a prerequisite before further assessing the role of phages. Two other disrupting agents were tested apart from temperature: sodium sulfite (50 to 400 mg/L), which is employed as a food preservative agent in the industry [31], and ethanol (5 to 15%), as it is generated in cider. No disturbance of the microbial equilibria was observed in the tested conditions with these two compounds, which mainly had a micro-bistatic effect (data not shown). Thus, a heat shock was used as a disruptive agent and was applied to the model community after 24 h of incubation at 25 °C. Twenty-four hours after the heat shock (i.e., 48 h from the beginning of the experiment), the microbial counts were 10^2^–10^4^ CFU/mL in conditions D and P for all the strains (just before phage addition), whereas they reached 10^6^–10^8^ CFU/mL in condition C (Figure 2A). Although 2-log variations were observed, these differences were not statistically different in the short term after heat shock, probably due to the use of non-parametric statistics. Nevertheless, the mead-term significant differences (*p*-value < 0.05) between conditions C and D were shown at 96 h for strain *S. uvarum* UCMA 10446 and *O. oeni* UCMA 19297, and at 168 h for *Ln. mesenteroides* CIM 3004 and *O. oeni* UCMA 19297 (Table S1), thus, confirming that a perturbation of the community occurred.

After 72 h, the heat shock was performed at 15 °C, so that beforehand, the strains could reach similar cellular levels (≈10^5^ CFU/mL) to those obtained at 25 °C (Figure 2B). Twenty-four hours after the heat shock (96 h from the beginning of the experiment), the microbial concentrations ranged between 10^6^ and 10^8^ CFU/mL in conditions C and between 10^1^ and 10^4^ CFU/mL in condition D and P. Again, although wide variations were observed, a significant difference was shown between conditions C and D only for the strain *S. uvarum* UCMA 10446 at 96 h (*p*-value = 0.02) (Table S2), confirming a real but more limited disturbance at this temperature.

This perturbation of the microbial equilibria in the model microbial community being established, it was possible to continue the experiment with the addition of phages in aliquot P, and to continue monitoring the behavior of the microbial model.

#### 3.1.2. Phages Contributed to Restoring Microbial Equilibria at 25 °C

The second step of the work was to assess the differences between the different conditions, in order to determine the effect of the addition of phages to the model microbial community (condition P).

In condition C at 72 h and 96 h, *L. mali* UCMA 16447, *Ln. mesenteroides* CIM 3004 and *O. oeni* UCMA 19297 co-dominated, reaching 10^8^-10^9^ CFU/mL, while *S. uvarum* UCMA 10446 was sub-dominant (10^7^ CFU/mL). At 168 h, *Ln. mesenteroides* CIM 3004 and *S. uvarum* UCMA 10446 decreased, while *L. mali* UCMA 16447 and *O. oeni* UCMA 19297 remained stable and co-dominant (Figure 2A).

In the condition subjected to disturbance without phage (D), at 72 h (2 days after the heat shock), the *L. mali* UCMA 16447 and *Ln. mesenteroides* CIM 3004 strains were dominant (10^7^–10^8^ CFU/mL at 72 h and at 10^8^–10^9^ CFU/mL at 96 h), whereas the *O. oeni* UCMA 19297 and *S. uvarum* UCMA 10446 strains remained sub-dominant, with counts around 10^5^–10^6^ CFU/mL at 72 h and 96 h (Figure 2A), and were significantly different from condition C (*p*-values < 0.05, Table S1). These levels remained similar at 168 h.

In condition P, the cellular levels of the four strains were not significantly different from those of condition D, except for *Ln. mesenteroides* CIM 3004 at 96 h, showing that phage addition reduced the cellular levels of one of the target LABs and modified the equilibria. Compared to condition C, phage addition was associated to significantly lower cellular levels at 72 h, with *Ln. mesenteroides* CIM 3004 around 10^4^ CFU/mL (*p*-value = 0.02 at 72 h) (Table S1), and the other strains around 10^5^ CFU/mL (*p*-values < 0.05, Table S1). At 96 h, only a significant difference for *L. mali* UCMA 16447 between conditions C and P was still observed (*p*-value = 0.02, Table S1). For the other strains at this point, and for all four strains at 168 h, microbial dynamics were similar in conditions C and P. *L. mali* UCMA 16447 and *O. oeni* UCMA 19297 co-dominated at the end, while *Ln. mesenteroides* CIM 3004 and *S. uvarum* UCMA 10446 were sub-dominant, and no significant differences were observed (*p*-values > 0.05) (Figure 2A, Table S1). Phage dynamics increased over time to reach 10^9^ PFU/mL for phage UCMA 21115 and 10^8^ PFU/mL for phage PLE001 at 168 h.

The pH evolution was quite different in condition P compared to conditions C and D. The final pH was 4.5 ± 0.5 for condition P, while it was 4.0 ± 0.01 for condition C and 3.90 ± 0.01 for condition D.

To summarize, the microbial equilibria in the condition with phages (P) were closer to those of the control condition (C) than to those of the condition without phages (D) at 25 °C, especially at 96 h and 168 h. Indeed, in conditions C and P, the microbial levels were the same overall, while in condition D, the *L. mali* UCMA 16447 and *Ln. mesenteroides* CIM 3004 strains were dominant. Overall, it seemed that at 25 °C, the two phages targeting the *L. mali* UCMA 16447 and *Ln. mesenteroides* CIM 3004 strains contributed to compensating for the effects of the heat-shock disturbance on the model microbial community.

#### 3.1.3. Phages Enhanced Disturbance at 15 °C

At 15 °C in condition C, all the strains had similar cellular levels and co-dominated over time, and all reached about 10^8^ CFU/mL at 220 h with a slight decrease in the *S. uvarum* UCMA 10446 strain at this time point (10^7^ CFU/mL) (Figure 2B).

In the condition subjected to disturbance without phage (D), although no significant differences (*p*-values > 0.05, Table S2) in microbial counts were observed between conditions C and D over time, except for the *S. uvarum* UCMA 10446 strain at 96 h, the *O. oeni* UCMA 19297 and *S. uvarum* UCMA 10446 strains appeared as sub-dominant in condition D from 144 h (Figure 2B).

The addition of phages in condition P disturbed the microbial equilibria in the community, which was especially visible for the *Ln. mesenteroides* CIM 3004 strain. At 144 h, *Ln. mesenteroides* CIM 3004 was reduced to 10^3^ CFU/mL. It was no longer detected (detection threshold of 10^1^ CFU/mL) from 168 h and thereafter in condition P, which contrasted with conditions C and D (Figure 2B). Since null values create a false result of the non-parametric statistic tests, statistical significance was not assessed for this strain at 168 h and 220 h between condition P and conditions C and D. Nonetheless, these observations showed the influence of phages targeting this strain. The *L. mali* UCMA 16447 strain seemed less impacted by the presence of its phages than the *Ln. mesenteroides* CIM 3004 strain at this temperature. However, *L. mali* UCMA 16447 increased slowly over time and remained at a level lower than 10^7^ CFU/mL (Figure 2B). No significant differences (*p*-values > 0.05, Table S2) were observed for this strain between condition P and conditions C and D. Regarding phage dynamics, phages UCMA 21115 and PLE001 increased until 168 h, reaching 10^8^ PFU/mL and 10^9^ PFU/mL, respectively (Figure 2B). A decrease to 10^6^ PFU/mL and 10^5^ PFU/mL for phage UCMA 21115 and phage PLE001, respectively, was then observed at 220 h (Figure 2B).

The pH values remained around 5.2 ± 0.12 after 72 h and during all the experiment in conditions C and P, whereas in condition D, the pH dropped from 5.2 ± 0.3 to 4.3 ± 0.04 at 220 h (Figure 2B).

To summarize, microbial levels in the condition with phages (P) were distant from the control condition (C) and the condition without phages (D), at 15 °C. In condition P, the microbial levels were unbalanced with the lack of the strain *Ln. mesenteroides* CIM 3004, which was undetectable, contrary to conditions C and D. Overall, at 15 °C, the presence of the phages impacted the microbial equilibria and seemed to worsen the microbial imbalance caused by the heat-shock disturbance.

### 3.2. The Metabolic Signatures Followed the Same Patterns as Microbial Enumerations

Thirty-two compounds including glucose, fructose, ethanol, lactic acid, acetic acid and citric acid, which were analyzed by HPLC, and 26 volatile organic compounds (VOCs), which were identified by GC-MS, were monitored (Table S3).

#### 3.2.1. Phages Partly Restored the Metabolic Signature of the Community after Disturbance at 25 °C

A Principal Component Analysis (PCA) was performed using HPLC and GC-MS data for all the time points (from T0 to T168 h) (Figure S2). Overall, the samples were distributed according to their analysis time rather than the treatment performed (C, D or P). The first sampling times (C48, D48, P48, C72, D72 and P72) were associated to high concentrations of glucose, fructose and citric acid, whereas the last sampling times (C96, D96, P96, C168, D168 and P168) were associated to a high value of VOCs (Figure S2). As the majority of VOCs were detected at the last sampling points (after 96 h), PCA was performed at 96 h, and the two principal component dimensions (Dim 1 and Dim 2) accounted for 84.47% of the total variance (49.80% and 34.67% for Dim 1 and Dim 2, respectively) (Figure S3). According to the selected criteria (contribution ≥ 3 and cos^2^ ≥ 0.5 for Dim 1 and Dim 2), 27 variables were displayed on Dim 1 and Dim 2, and the samples were clearly separated into three distinct groups (C, D and P). Condition C was characterized by a high abundance of organic acids (lactic and acetic acids), fatty acids (hexanoic acid) and ethanol, and by a low abundance of sugars (fructose and glucose) and alcohols (2-methylbutanol) (Figure S3). Condition D was characterized by a high abundance of aldehydes (nonanal, phenylacetaldehyde) and esters (isoamyl acetate), and by a low abundance of fatty acids (hexanoic acid, butanoic acid) (Figure S3). Condition P was distinguished by a high abundance of esters (methyl lactate, ethyl octanoate), sugars (glucose, fructose) and alcohols (isoamyl alcohol, 2-phenylethanol), and by a low abundance of organic acids (lactic and acetic acids) and ethanol (Figure S3).

**Figure 3:**
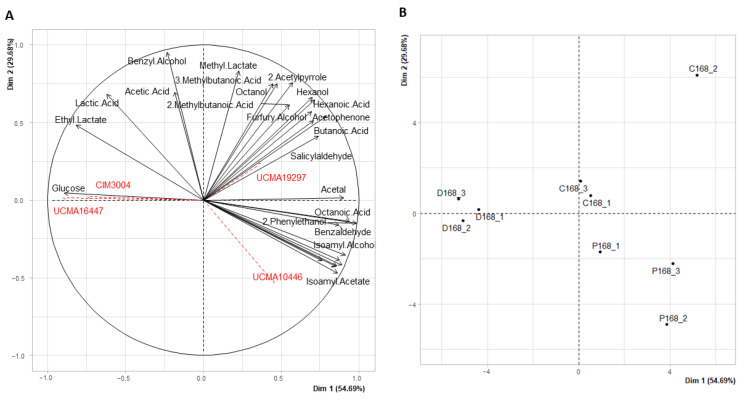
(**A**) Principal Component Analysis of the studied compounds (black arrows) with illustrative variables representing the four strains of the model microbial community (red arrows). (**B**) Distribution of the samples in condition C (control), D (disturbance without phages) and P (disturbance with phages) at 168 h for the experiment at 25 °C. Samples are named as follows: Condition (C: control, D: disturbance without phages, P: disturbance with phages). Time point (0, 48 h, 72 h, 96 h, 168 h). Biological replicate (1, 2, 3) (e.g., C168_1 means Control at 168 h, biological replicate 1).

In the same way, PCA was displayed at 168 h where Dim 1 and Dim 2 accounted for 84.37% of the total variance (54.69% and 29.68% for Dim 1 and Dim 2, respectively) (Figure 3).

According to the selected criteria (contribution ≥ 3 and cos^2^ ≥ 0.5 for Dimensions 1 and 2), 27 variables were selected for this PCA analysis. Condition D was separated from conditions C and P, themselves being close to each other on Dim 1 but more distant on Dim 2. Condition D was characterized by higher abundances of ethyl lactate, lactic acid and glucose, and by lower abundances of fatty acids (octanoic acid) and alcohols (2-phenylethanol) than the other conditions (Figure 3). Replicates from conditions C and P were slightly separated from each other on Dim 1 and Dim 2 (C168_1 and C168_3 from C168_2 for condition C, and P168_2 and P168_3 from P168_1 for condition P), but remained grouped when compared to condition D. Indeed, conditions C and P were represented by higher abundances of fatty acids (hexanoic acid, butanoic acid, octanoic acid), alcohols (hexanol, isoamyl alcohol, 2-phenylethanol) and esters (isoamyl acetate, methyl lactate) than condition D (Figure 3).

**Figure 4:**
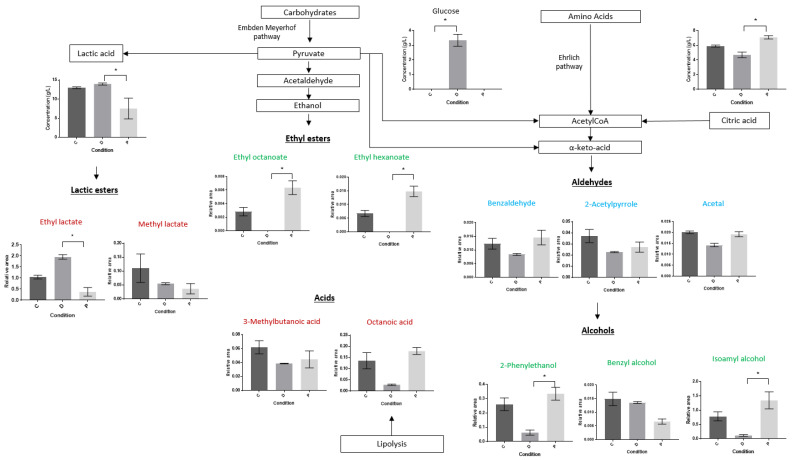
Principal compounds and hypothetical representation of the metabolic pathways potentially involved in the production/consumption of these compounds at 25 °C between conditions C, D and P at 168 h. Green compounds were principally associated to yeast activity, red compounds to LAB activity and blue compounds to yeast and/or LAB activity. Concentrations of lactic acid and citric acid were determined by HPLC and expressed in g/L. The volatile compounds were analyzed by GC-MS and expressed with relative area (standardized with internal standards). A Kruskal–Wallis test was performed for each compound, and multiple comparisons were performed using Dunn’s procedure. A *p*-value of ≤ 0.05 was considered significant (*).

In order to observe the differences in the abundances of the most involved compounds in the PCA, compounds with a contribution value of ≥ 5 and a cos^2^ value of ≥ 0.5 for Dim 1 and Dim 2 were selected at the final sampling time (168 h). A total of 15 compounds were, thus, retained (Figure 4).

Among these compounds, only those analyzed by HPLC (lactic acid, citric acid, glucose) were quantified in grams per liter, while the others, which were identified by GC-MS, were expressed in relative area (standardized with internal standard). Overall, the abundance levels between conditions C and P were close compared to condition D. For example, esters that were principally associated to yeast activity—such as ethyl octanoate, ethyl hexanoate and alcohols derived from amino acid degradation such as 2-phenylethanol—showed significant differences (*p*-value < 0.05) between conditions P and D, with abundances higher in condition P than in condition D, and, as such, closer to the abundances of condition C (Figure 4). Even if no significant statistical differences were found between conditions D and C, the abundance levels were still higher in condition C than in condition D for these compounds (Figure 4). For example, the relative area of 2-phenylethanol was 0.3 in condition P, 0.25 in condition C and 0.05 in condition D (Figure 4). The same trends were observed for citric acid and isoamyl alcohol, for which the abundance levels were significantly higher in condition P than in condition D (*p*-value < 0.05, Figure 4). The concentration of citric acid was of 7 g/L in P, 6 g/L in C and 4 g/L in D, and the relative area of isoamyl alcohol was 1.3 in condition P, 0.8 in condition C and 0.1 in condition D (Figure 4). The same tendencies were observed for fatty acids probably originating from lipolysis or β-oxidation (octanoic acid), and for aldehydes (benzaldehyde, acetal), even if no significant difference was found (*p*-value > 0.05, Figure 4). Concentrations of lactic acid and its ester, ethyl lactate, which is mostly associated to LAB activity, were significantly higher in condition D than in condition P, and the concentration of glucose was significantly higher in condition D than in condition C (*p*-value < 0.05, Figure 4). For example, the relative area of ethyl lactate was 2 in condition D, while it was 0.5 and 1 in condition P and condition C, respectively. No significant differences were observed for compounds such as 2-acetylpyrrole, 3-methylbutanoic acid, methyl lactate and benzyl alcohol between the three conditions (*p*-value > 0.05, Figure 4).

To summarize, the metabolic signatures of the control condition (C) and the condition with phages (P), at 25 °C and after 168 h, were close, with a higher abundance in volatile organic compounds such as esters (ethyl octanoate, ethyl hexanoate) and alcohols (2-phenylethanol) than in the condition without phages (D). This condition was characterized by higher levels of lactic acid and its ester ethyl lactate, presumably reflecting a higher activity of the LAB strains, and a lower activity of the yeast strain than in conditions C and P, at 25 °C. Overall, the presence of phages, in addition to contributing to restoring the microbial equilibria, also influenced the metabolic signature of the condition where they were present.

#### 3.2.2. The Metabolic Signature of Condition P Was Different from Conditions C and D at 15 °C

**Figure 5:**
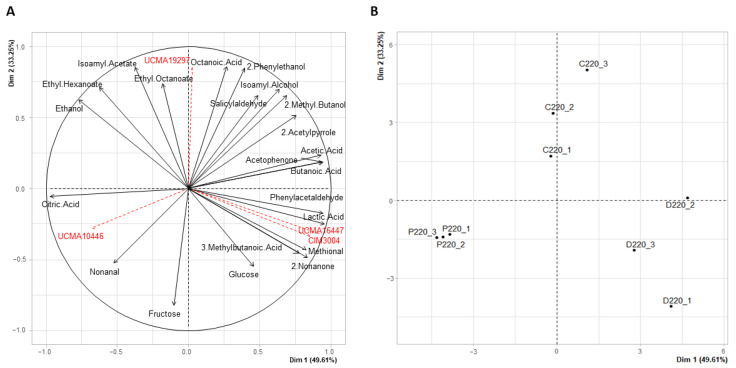
(**A**) Principal Component Analysis of the studied compounds (black arrows) with illustrative variables representing the four strains of the model (red arrows). (**B**) Distribution of the samples in condition C (control), D (disturbance without phages) and P (disturbance with phages) at 220 h for the experiment at 15 °C. Samples are named as follows: Condition (C: control, D: disturbance without phages, P: disturbance with phages). Time point, 220 h. Biological replicate (1, 2, 3) (e.g., C220_1 means Control at 220 h, biological replicate 1).

At 15 °C, PCA was also displayed using time points from T0 to T220 h (Figure S4). As at 25 °C, the samples were distributed according to time rather than condition (C, D or P). The first sampling times (0, 96 h and 144 h) were associated to high concentrations of sugars (glucose, fructose) and citric acid, and the last sampling time (220 h) was associated to VOCs in high abundance. For this time point, little differences between replicates from the three conditions were observed (Figure S4). A PCA was performed at the last time point (220 h), where Dim 1 and Dim 2 represented 82.86% of the total variance (49.61% and 33.25% for Dim 1 and Dim 2, respectively) (Figure 5).

Among the 32 identified compounds, 22 were retained for further analyses, as described above and according to the selected criteria (contribution ≥ 3 and cos^2^ ≥ 0.5 for Dim 1 and Dim 2). Condition P was separated from conditions C and D and presented a higher abundance of citric acid and aldehydes (nonanal) than conditions C and D. Condition C was characterized by high levels of alcohols (2-phenylethanol, isoamyl alcohol, 2-methylbutanol), and condition D by high levels of fatty acids (octanoic acid, butanoic acid, 3-methylbutanoic acid) (Figure 5).

Compounds with a contribution value of ≥ 5 and a cos^2^ value of ≥ 0.5 for Dim 1 and Dim 2 were selected at the final sampling time (220 h) at 15 °C (Figure S5). Differences in abundance levels between conditions C, D and P were less marked than in the experiment at 25 °C. Nevertheless, significant differences between conditions D and P were found for compounds such as aldehydes (phenylacetaldehyde, methional), organic acids (lactic, citric and acetic acids) and fatty acids (butanoic acid), with higher values in condition D than in condition P, excepted for citric acid, reflecting a higher activity of LAB (*p*-values < 0.05, Figure S5). No significant differences between conditions C and P were observed for these compounds (*p*-values > 0.05, Figure S5). For example, the relative area of phenylacetaldehyde was 0.16 in condition D, while it was 0.02 in condition P and 0.09 in condition C (Figure S5). The concentration of lactic acid was also 80 g/L in condition D, whereas it was 5 g/L in condition P and 30 g/L in condition C (Figure S5). The abundance levels in conditions C and D were close for some compounds, such as acids (lactic and acetic acids), alcohols (2-phenylethanol), aldehydes (phenylacetaldehyde, methional and acetylpyrrole) and fatty acids (butanoic acid), compared to those in condition P, except for some compounds which fluctuated, such as esters (ethyl octanoate, ethyl hexanoate) or ketone (2-nonanone) (Figure S5).

To summarize, the metabolic signature of the condition without phage (D) was different from the control condition (C) and the condition with phages (P) at 15 °C, with a less marked variety of metabolic compounds than for the other conditions. Overall, the presence of phages also influenced the metabolic signature at 15 °C and enhanced the microbial imbalance after the disturbance.

## 4. Discussion

Model microbial communities are often used for industrially-oriented research, for example, to enhance the metabolism of microbial strains in order to produce compounds of interest. For example, these microbial models have been developed for the production of bacterial cellulose-based composite materials [32], or for the biosynthesis of butyl acetate by co-cultures of *Clostridium acetobutylicum* and *Actinobacillus succinogenes* [33]. These model microbial communities can also be used to obtain knowledge about microbial interactions in nature [34]. Model microbial communities that take phages into consideration are still to be developed, even if first attempts have been made, especially for studying the intestinal gut microbiota [35]. For example, after microbiota dysbiosis in mice caused by antibiotic treatment, commensal phage transplantations contributed to reshaping the microbial equilibria of the microbiota to a state close to that observed before the antibiotic treatment [9]. The role of phages in fermented foods and beverages is little described on the scale of microbial communities taking into account the interactions between phages and bacteria [36,37]. To our knowledge, the literature on model microbial communities integrating phages is not available for the study of microbial interactions in fermented foods and beverages. In this context, it was interesting to monitor the behavior of a model microbial community that was inspired by a fermented beverage (cider), which included phages, in order to understand their potential role in microbial dynamics, especially when the microbial equilibria were disturbed (creation of a disturbance). The model microbial community included four microbial strains (three LAB and a yeast strain) belonging to genera and species retrieved in cider, and two phages targeting the *L. mali* UCMA 16447 and *Ln. mesenteroides* CIM 3004 strains of this microbial community model. The experiments were performed in MRS medium, which was supplemented with fructose, the main sugar in cider. As it was the first model of this type, synthetic medium was preferred to sterile cider in order to better standardize the cellular levels and phage activity, and thus, to better visualize phage–bacteria interactions. Indeed, bacterial growth and phage stability are more fastidious and less reproducible in the cider matrix, which would have certainly blurred the monitoring of the community. The impact of phages on the model microbial community varied depending on the temperature of incubation. At 25 °C, the phages seemed to help restore the microbial equilibria after disturbance caused by a heat shock, the microbial dynamics in the presence of phages (P) being closer to those of the control condition (C) than of the community without phages (D). In the model microbial community, the disturbance caused by the heat shock at 50 °C unintentionally led to the dominance of the two strains targeted by phages (*L. mali* UCMA 16447 and *Ln. mesenteroides* CIM 3004). The presence of phages was permitted to approximately restore their cellular levels to the same levels as in the control condition. The temperature is known to affect both phage activity and bacterial physiology. This could explain the differences between the two tested conditions. This has already been observed for *Weissella* phages from kimchi, which were more stable and active at 7 °C than at 30 °C [38]. The *Ln. mesenteroides* CIM 3004 strain may also be in a physiological state that allows it to better resist phage infection at 25 °C than at 15 °C. This phenomenon has already been observed in a study about the *Leuconostoc* and *Weissella* strains from kimchi, which were co-cultivated at 7 °C in the presence of their phages. In this case, the phages destroyed their hosts, suggesting that the *Leucosnostoc* strains were sensitive to phage attack at low temperatures [39]. The dynamics were different concerning phage UCMA 21115 targeting the *L. mali* UCMA 16447 strain. At 15 °C and 25 °C, phage UCMA 21115 did not significantly affect its hosts in the view of the enumeration results. Such a phenomenon has already been demonstrated for Swiss hard cheese starter cultures, where two highly abundant *Streptococcus* phages co-existed in the starter community without any negative impact on host growth and persistence [40]. In this last study, phages persisted despite the presence of CRISPR spacers within the bacterial population, suggesting that they could evade the CRISPR-based immunity. For the *L. mali* UCMA 16447 strain, no CRISPR locus was identified within its genome, suggesting that other mechanisms that still need to be deciphered occurred to resist the lytic phage UCMA 21115, which was present at high concentrations (about 10^9^ PFU/mL at 168 h in the experiment at 25 °C).

Phages have been shown to affect the host bacteria, leading to cascading effects on other bacterial species and, as a consequence, on the gut metabolome in a mouse model [35]. The presence of phages targeting *L. mali* UCMA 16447 and *Ln. mesenteroides* CIM 3004 led to a variation in the *O. oeni* UCMA 19297 and *S. uvarum* UCMA 10446 strains, which were not targeted by the phages. In order to observe whether the phages could also influence the metabolic signature of the current study’s model microbial community, the production of organic acids and volatile organic compounds was monitored and visualized using PCA analyses. As observed for the microbial dynamics, metabolic trends were similar between the control condition (C) and the condition with phages (P) at 25 °C, whereas the condition without phages (D) differed. At 15 °C, the metabolic composition in condition P was different from conditions C and D. For example, ethyl lactate, which is associated to LAB activity, was highly predominant in condition D at 25 °C, where the *L. mali* UCMA 16447 and *Ln. mesenteroides* CIM 3004 strains were predominant. When in excess, this compound is often undesirable in fermented beverages, particularly in cider, as it causes olfactory defects [41]. On the contrary, conditions C and P showed high contents of esters (ethyl octanoate, ethyl hexanoate) and aromatic compounds such as 2-phenylethanol, which are often associated to yeast activity, and generated fruity notes and good perceptions in fermented beverages [42]. These results are in agreement with microbial counts, because the *S. uvarum* UCMA 10446 strain was at high concentrations in conditions C and P at 25 °C. Regarding the experiment at 15 °C, condition P was characterized by high contents in citric acid compared to conditions C and D. Citric acid metabolism is often associated to LAB activity, and *Ln. mesenteroides* is especially studied for its ability to metabolize citric acid [43]. The high level of citric acid and the lower level of volatile organic compounds in condition P could be explained, in part, by the absence of the *Ln. mesentoides* CIM 3004 strain. Deciphering the contribution of each micro-organism to the consumption and production of specific compounds is not easy, as LABs, in particular, share common enzymes that are implicated in several metabolic pathways. To complete the current study and delve deeper into the metabolome analysis in relation to the presence of phages, it would be interesting, for example, to perform metatranscriptomic assays on the community, as it was performed in Swiss-type Maasdam cheese during ripening to associate the production of flavor compounds to each strain used in a cheese community model [44]. Metabolomic approaches enable the study of huge numbers of metabolites in a sample, and thus, could be another way to more precisely characterize the model with very specific metabolic signatures of the different conditions. Metabolomics has already been performed on fermented food products, such as for the characterization of new recipes of beer [45], to characterize fermented milk with different probiotics [46] and to identify three different teas, thanks to their metabolites [47]. These metabolomic techniques also enabled observation of the impact of a bioprotective strain of *Metschnikowia pulcherrims* on the volatile composition of different wines [48]. Thus, the use of these methods could allow to more precise information to be obtained about the impact of phages in more complex model microbial communities.

## 5. Conclusions

This is the first study regarding the impact of phages on a model microbial community inspired by cider. It showed that phages shape the microbial dynamics as well as the metabolism of the community, especially depending on the temperature, after a disturbance. Differences were observed regarding sugar consumption, organic acid production and volatile organic-compound production. This work provides information about the dynamics of phages–bacteria interactions in a model microbial community inspired by a fermented beverage and strengthens the idea of the important role played by phages in microbial communities in such a matrix, as they can directly and indirectly modulate both microbial populations and associated metabolites. A better understanding of this type of interaction in fermented foods is essential to provide information about the dynamics of microbial populations during the fermentation processes, and even so, for the sensorial properties of foods.

## Data Availability

The data presented in this study are available on request from the corresponding author.

## Supplementary Materials

The following supporting information can be downloaded at: https://www.mdpi.com/article/10.3390/v14102283/s1, Figure S1: Prophages induction with mitomycin C (MC) in *L. mali* UCMA 16447 (A), *Ln. mesenteroides* (B) and *O. oeni* UCMA 19297 (C). Green curve: negative control (without MC), blue curve: MC 0.1 µg/mL, and red curve: MC 1 µg/mL. Error bars were obtained from 4 technical replicates, and optical density at 600 nm was measured using a microplate reader (TECAN SPARK). Figure S2: (A) Principal Component Analysis of the studied compounds (black arrows) with illustrative variables representing the four strains of the model (red arrows). (B) Distribution of the samples in condition C (control), D (disturbance without phages) and P (disturbance with phages) for all times at 25 °C. Figure S3: (A) Principal Component Analysis of the studied compounds (black arrows) with illustrative variables representing the four strains of the model (red arrows). (B) Distribution of the samples in condition C (control), D (disturbance without phages) and P (disturbance with phages) at 96 h for the experiment at 25 °C. Figure S4: (A) Principal Component Analysis of the studied compounds (black arrows) with illustrative variables representing the four strains of the model (red arrows). (B) Distribution of the samples in condition C (control), D (disturbance without phages) and P (disturbance with phages) for all times at 15 °C. Figure S5: Principal compounds and hypothetical representation of the metabolic pathways potentially involved in the production/consumption of these compounds at 15 °C between conditions C, D and P at 220 h. Table S1: Kruskal–Wallis and Dunn’s multiple comparison analysis results of microbial enumerations for the experiment at 25 °C. Table S2: Kruskal–Wallis and Dunn’s multiple comparison analysis results of microbial enumeration for the experiment at 15 °C. Table S3: Volatile compounds identified and analyzed by GC-MS during the study.
