# Supplementary material for: Phages Shape Microbial Dynamics and Metabolism of a Model Community Mimicking Cider, a Fermented Beverage
Source: Viruses. 2022 Oct 17;14(10):2283. doi: 10.3390/v14102283 (PMC9609687; doi:10.3390/v14102283)
Supplement: Supplementary file 1 [file viruses-14-02283-s001.zip › Figure S1..pptx]

## Slide 1
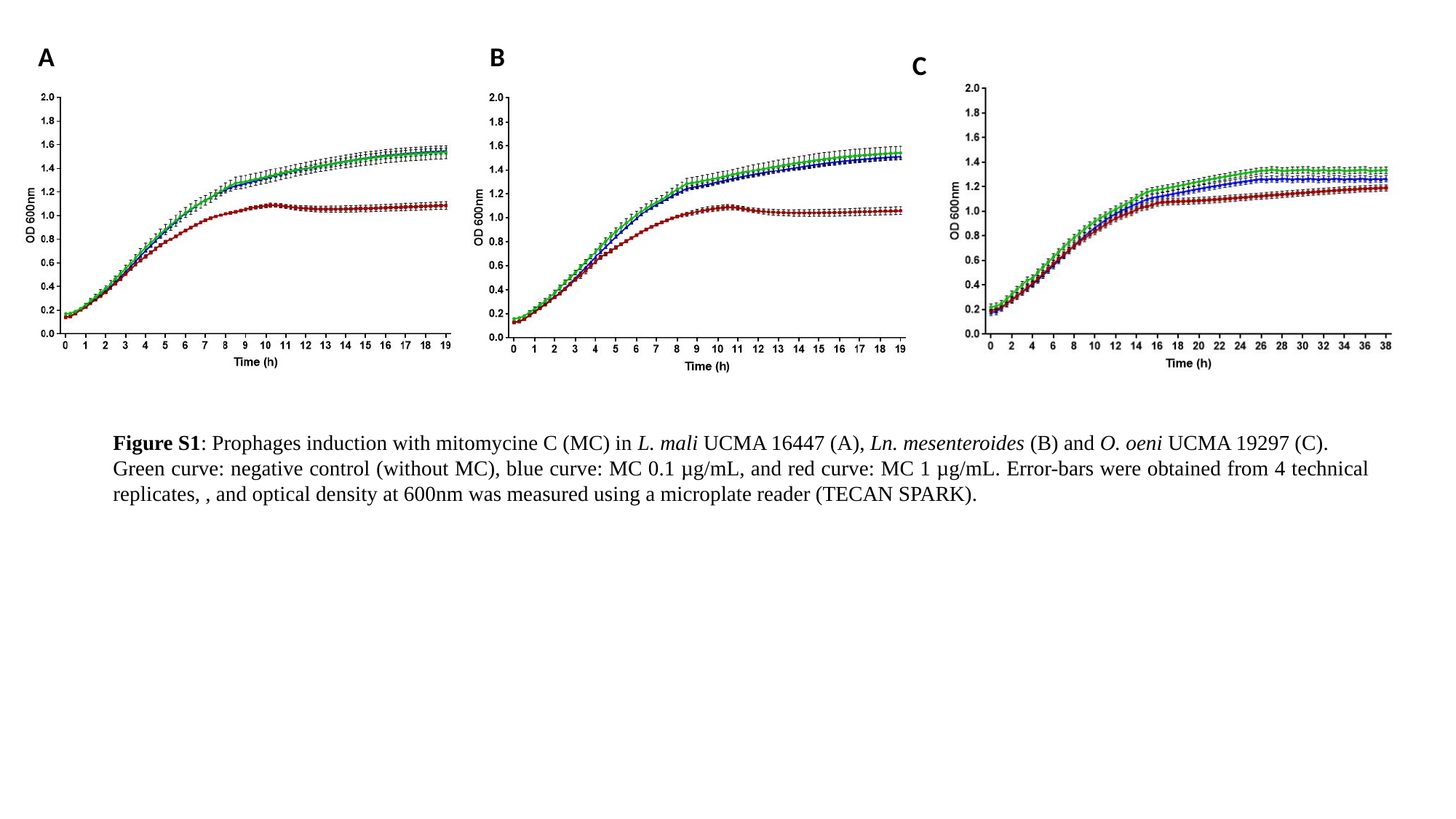

A
B
C
Figure S1: Prophages induction with mitomycine C (MC) in L. mali UCMA 16447 (A), Ln. mesenteroides (B) and O. oeni UCMA 19297 (C).
Green curve: negative control (without MC), blue curve: MC 0.1 µg/mL, and red curve: MC 1 µg/mL. Error-bars were obtained from 4 technical replicates, , and optical density at 600nm was measured using a microplate reader (TECAN SPARK).
