# Supplementary material for: Phages Shape Microbial Dynamics and Metabolism of a Model Community Mimicking Cider, a Fermented Beverage
Source: Viruses. 2022 Oct 17;14(10):2283. doi: 10.3390/v14102283 (PMC9609687; doi:10.3390/v14102283)
Supplement: Supplementary file 1 [file viruses-14-02283-s001.zip › Figure S2..pptx]

## Slide 1
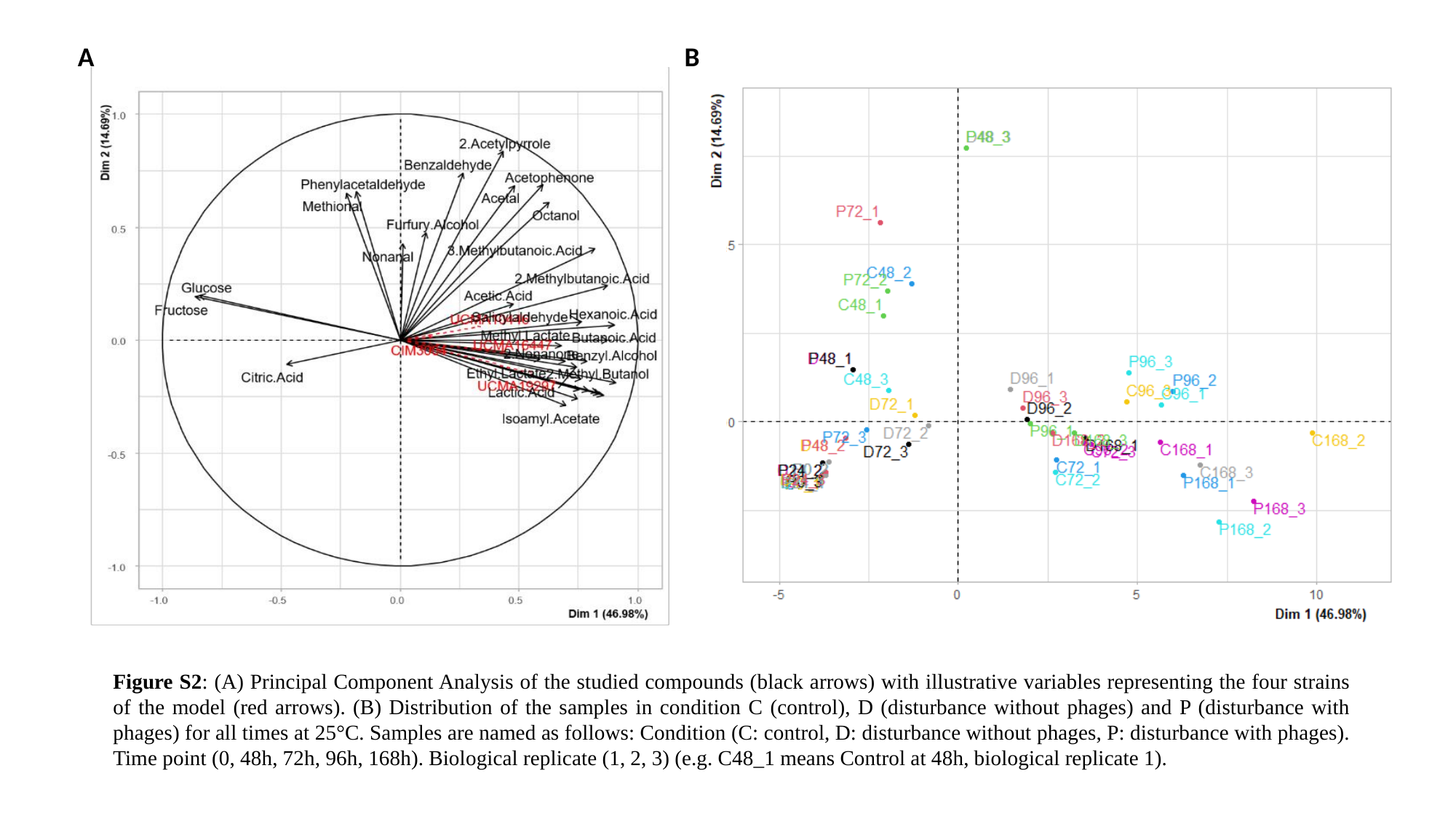

A
B
Figure S2: (A) Principal Component Analysis of the studied compounds (black arrows) with illustrative variables representing the four strains of the model (red arrows). (B) Distribution of the samples in condition C (control), D (disturbance without phages) and P (disturbance with phages) for all times at 25°C. Samples are named as follows: Condition (C: control, D: disturbance without phages, P: disturbance with phages). Time point (0, 48h, 72h, 96h, 168h). Biological replicate (1, 2, 3) (e.g. C48_1 means Control at 48h, biological replicate 1).
