# Supplementary material for: Phages Shape Microbial Dynamics and Metabolism of a Model Community Mimicking Cider, a Fermented Beverage
Source: Viruses. 2022 Oct 17;14(10):2283. doi: 10.3390/v14102283 (PMC9609687; doi:10.3390/v14102283)
Supplement: Supplementary file 1 [file viruses-14-02283-s001.zip › Figure S5..pptx]

## Slide 1
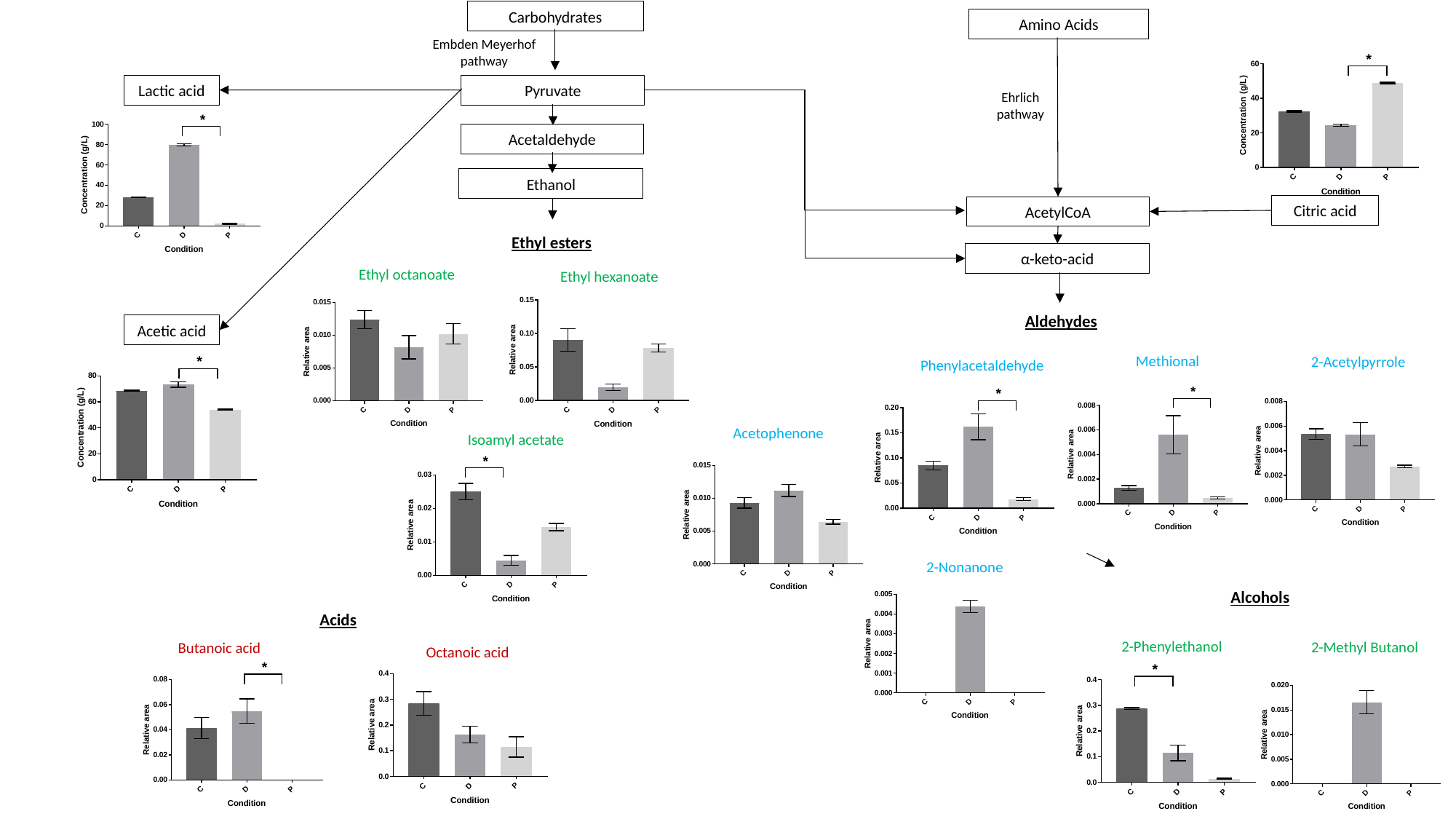

Carbohydrates
Amino Acids
Embden Meyerhof pathway
Lactic acid
Pyruvate
Ehrlich pathway
Acetaldehyde
Ethanol
Citric acid
AcetylCoA
Ethyl esters
α-keto-acid
Ethyl octanoate
Ethyl hexanoate
Aldehydes
Acetic acid
Methional
2-Acetylpyrrole
Phenylacetaldehyde
Acetophenone
Isoamyl acetate
2-Nonanone
Alcohols
Acids
2-Phenylethanol
2-Methyl Butanol
Butanoic acid
Octanoic acid
Figure S5: Principal compounds and hypothetical representation of the metabolic pathways potentially involved in the production/consumption of these compounds at 15°C between conditions C, D and P at 220h. Green compounds were principally associated to yeast activity, red compounds to LAB activity and blue compounds to yeast and/or LAB activity. Concentrations of lactic acid, acetic acid and citric acid were determined by HPLC and expressed in g/L. The other volatile compounds were analyzed by GC-MS and expressed with relative area (standardized with internal standards). Kruskal Wallis test was performed for each compound and multiple comparisons were performed using Dunn’s procedure. A p-value ≤ 0.05 was considered significant (*).
