# Supplementary material for: Phages Shape Microbial Dynamics and Metabolism of a Model Community Mimicking Cider, a Fermented Beverage
Source: Viruses. 2022 Oct 17;14(10):2283. doi: 10.3390/v14102283 (PMC9609687; doi:10.3390/v14102283)
Supplement: Supplementary file 1 [file viruses-14-02283-s001.zip › Table S2.pptx]

## Slide 1
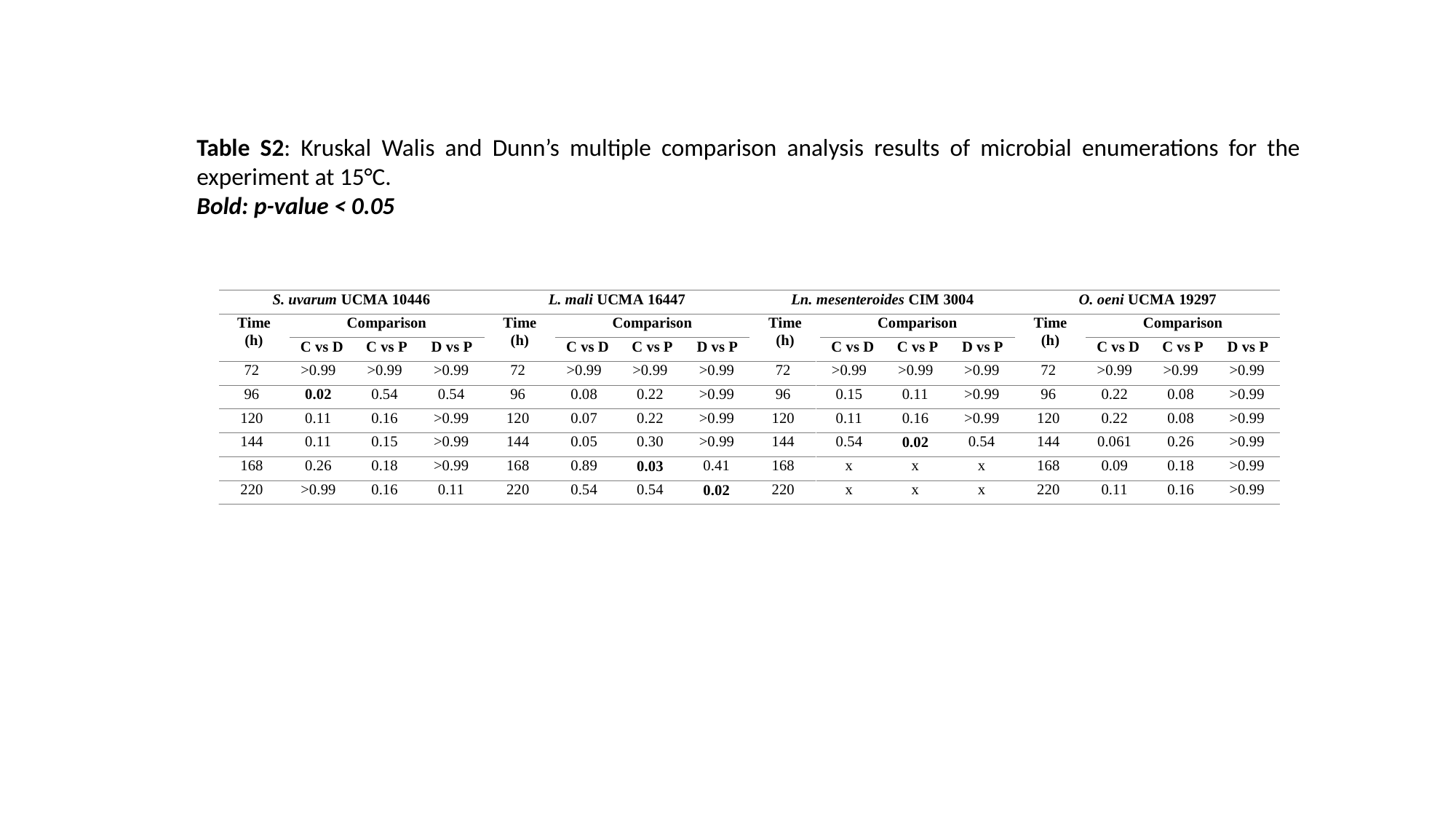

Table S2: Kruskal Walis and Dunn’s multiple comparison analysis results of microbial enumerations for the experiment at 15°C.
Bold: p-value < 0.05
