# Supplementary figures and images for: Phages Shape Microbial Dynamics and Metabolism of a Model Community Mimicking Cider, a Fermented Beverage
Source: Viruses. 2022 Oct 17;14(10):2283. doi: 10.3390/v14102283 (PMC9609687; doi:10.3390/v14102283)

## Slide 1
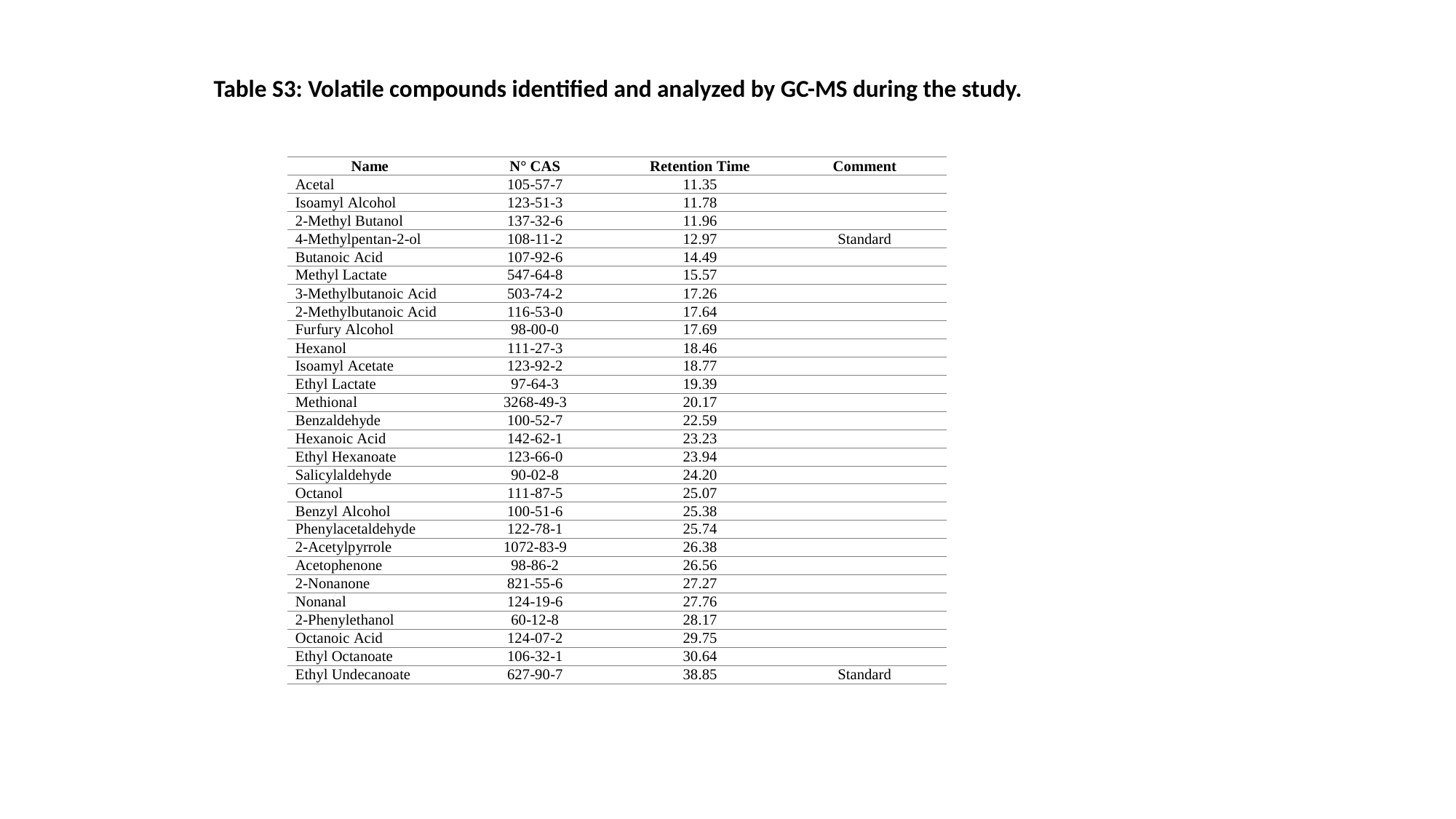

Table S3: Volatile compounds identified and analyzed by GC-MS during the study.

Supplement: Supplementary file 1 [file viruses-14-02283-s001.zip › Table S3.pptx]
